# Atorvastatin provides a new lipidome improving early regeneration after partial hepatectomy in osteopontin deficient mice

**DOI:** 10.1038/s41598-018-32919-9

**Published:** 2018-10-02

**Authors:** Maitane Nuñez-Garcia, Beatriz Gomez-Santos, Diego Saenz de Urturi, Daniela Mestre, Francisco Gonzalez-Romero, Xabier Buque, Virginia Gutiérrez-de Juan, María Luz Martinez-Chantar, Wing-Kin Syn, Olatz Fresnedo, Patricia Aspichueta

**Affiliations:** 1 Department of Physiology, Faculty of Medicine and Nursing, University of the Basque Country, UPV/EHU, Lejona, Spain; 2 Biocruces Health Research Institute, Barakaldo, Spain; 3 Liver disease Laboratory, Liver metabolism Laboratory, CIC bioGUNE, Centro de Investigación Biomédica en Red de Enfermedades Hepáticas y Digestivas (CIBERehd), Zamudio, Spain; 4 Division of Gastroenterology and Hepatology, Medical University of South Carolina, Charleston, South Carolina USA; 5 Section of Gastroenterology, Ralph H Johnson Veteran Affairs Medical Center, Charleston, South Carolina USA

## Abstract

Osteopontin (OPN), a multifunctional cytokine that controls liver glycerolipid metabolism, is involved in activation and proliferation of several liver cell types during regeneration, a condition of high metabolic demands. Here we investigated the role of OPN in modulating the liver lipidome during regeneration after partial-hepatectomy (PH) and the impact that atorvastatin treatment has over regeneration in OPN knockout (KO) mice. The results showed that OPN deficiency leads to remodeling of phosphatidylcholine and triacylglycerol (TG) species primarily during the first 24 h after PH, with minimal effects on regeneration. Changes in the quiescent liver lipidome in OPN-KO mice included TG enrichment with linoleic acid and were associated with higher lysosome TG-hydrolase activity that maintained 24 h after PH but increased in WT mice. OPN-KO mice showed increased beta-oxidation 24 h after PH with less body weight loss. In OPN-KO mice, atorvastatin treatment induced changes in the lipidome 24 h after PH and improved liver regeneration while no effect was observed 48 h post-PH. These results suggest that increased dietary-lipid uptake in OPN-KO mice provides the metabolic precursors required for regeneration 24 h and 48 h after PH. However, atorvastatin treatment offers a new metabolic program that improves early regeneration when OPN is deficient.

## Introduction

The liver has the capacity for regeneration after cellular damage or surgery resection. During regeneration, liver cells need to acquire sufficient energy and metabolic precursors to support the metabolic demands for rapid proliferation. Thus, a variety of metabolic pathways is switched on^1^. The synthesis of phosphatidylcholine (PC), the mayor phospholipid in cellular membranes, is coordinated with the activation of the cell cycle^2^ and is an essential step in cell proliferation^3,4^. However, the lack of CTP: phosphocholine cytidyltransferase in mice, the rate limiting enzyme responsible for producing nearly 70% of liver PC^5^, does not inhibit liver regeneration after partial hepatectomy (PH)^6^. Catabolism of PC generate compounds required for triacylglycerol (TG) synthesis^7^, which are major constituents of lipid droplets and accumulate transiently in hepatocytes after PH. It has been proposed that the hypoglycemia that follows PH induces systemic lipolysis providing the required fatty acids (FA) for hepatic lipogenesis^8^. This process will lead to rapid storage of intracellular TG within the regenerating liver, thus inducing a transient regeneration-associated steatosis^9^.

Although multiple studies have analyzed the expression patterns and functions of cytokines immediately after PH, the specific roles of each of these factors in the regulation of liver metabolic fluxes during regeneration remains poorly understood^1,10,11^. Osteopontin (OPN) is a multifunctional cytokine that is expressed in various tissues^12,13^. It plays an important role in obesity^12,14^ and in fibrogenesis during non-alcoholic steatohepatitis (NASH) progression^15^. It also modulates the activation and proliferation of several cell types during regeneration^16^. Recently, we reported that OPN controls *de novo* FA synthesis in hepatocytes and that liver PC concentration is regulated by circulating OPN^17^. In fact, high levels of OPN increase liver PC concentration, while PC concentration is low in OPN deficient mice^17^. We also found that inhibiting *de novo* cholesterogenesis, which is increased in OPN-KO mice liver, results in the restoration of PC concentration in OPN-KO mice, exemplifying the cross-talk between these metabolic pathways^17^. In this study, we investigated the roles of OPN in modulating liver TG and PC concentrations during liver regeneration after PH and the effects of atorvastatin on the regenerative liver lipidome in OPN deficient mice. The current study showed that serum OPN levels are increased 24 h after PH in WT mice, and that OPN deficiency led to alterations in the remodeling of PC and TG species during the first 24 h after PH. OPN deficiency had minimal effects on liver regeneration. We found that changes in quiescent liver lipidome in OPN-KO mice were associated with higher lysosome TG hydrolytic activity that was maintained 24 h after PH (but increased in WT mice). In sum, despite changes in PC concentration and PC and TG composition with OPN deficiency, remodeling of the liver lipid metabolism provides the metabolic precursors necessary for liver regeneration after PH. Treatment with atorvastatin ushered a new metabolic program that increased liver PC concentrations in OPN-KO mice 24 h after PH and promoted liver regeneration in these mice.

## Results

### OPN modulates liver TG and PC metabolism during liver regeneration after partial hepatectomy

**Figure 1:**
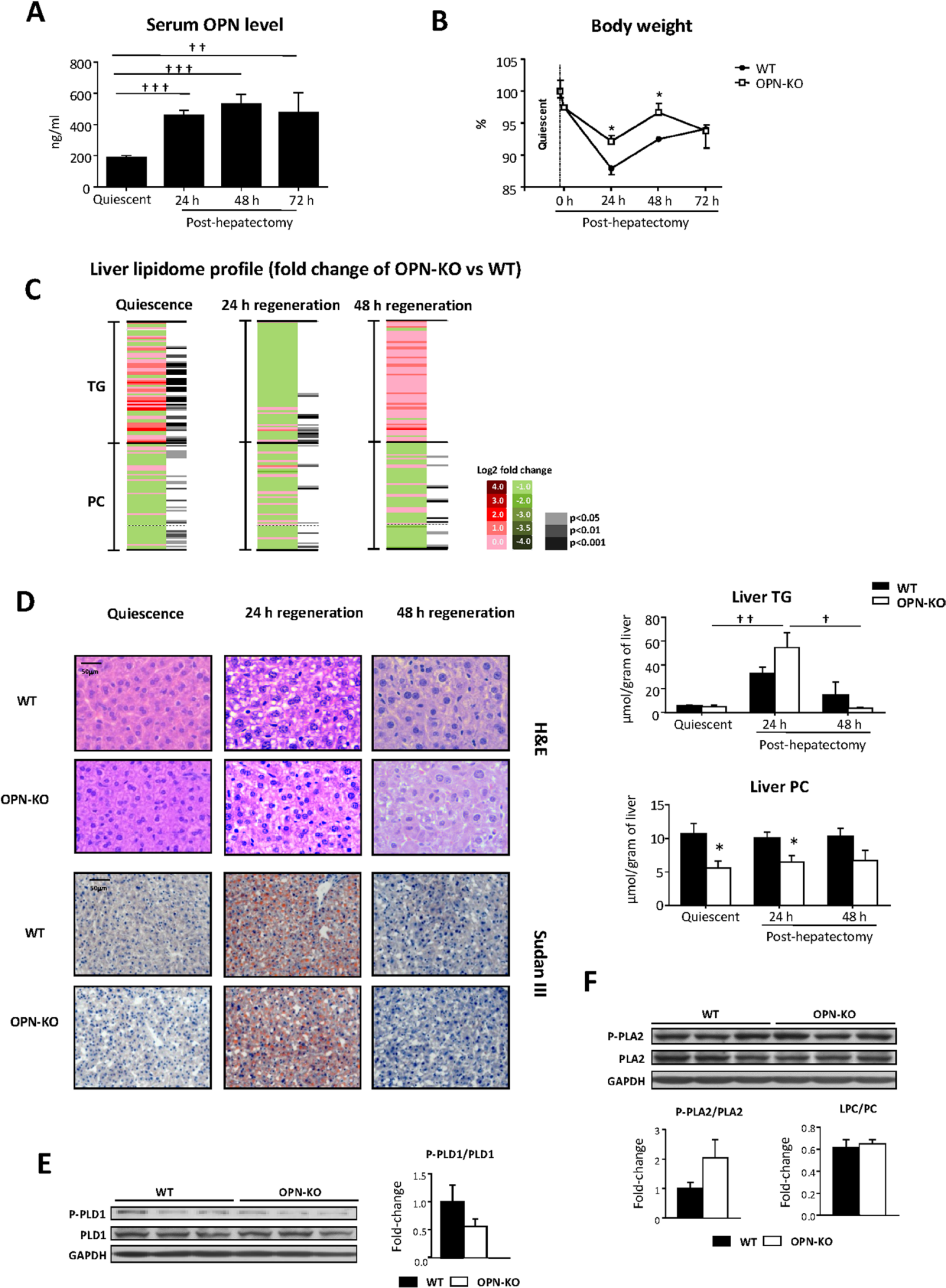
Modulation of liver lipidome after partial hepatectomy in OPN-KO mice. Osteopontin Knockout (OPN-KO) mice and their controls (WT) were subjected to partial hepatectomy (PH), and blood and liver were collected before and 24 h, 48 h and 72 h post-PH. (**A**) Serum OPN was quantified by ELISA in WT mice. (**B**) Animal weight, before PH and during liver regeneration (0 h, 24 h, 48 h and 72 h), is represented and the values are expressed as the percentage of the initial weight (100%). (**C**) Heat map representation of triacylglycerol (TG) and phosphatidylcholine (PC) metabolite subspecies abundance. In red, the increased lipids, in green the decreased lipids, in grays, the significant differences. (**D**) Representative liver sections stained with H&E (up) and Sudan III (down) and liver TG and phosphatidylcholine (PC) content is represented in nmol/gram of liver (right). (**E**) Phosphorylated (Thr-147) phospholipase D1 (P-PLD1) and total phospholipase D1 (PLD1) protein levels were assessed by immunoblotting. (**F**) Calcium free posphorylated (Ser-505) phospholipase A2 (P-PLA2) and total phopholipase A2 (PLA2) protein levels were assessed by immunoblotting.The LysoPC (LPC) to PC ratio was measured. In all the immunoblots glyceraldehyde-3-phosphate dehydrogenase (GAPDH) was used as a loading control. Values are mean ± SEM from 5–8 animals per group. Significant differences are denoted by *p < 0.05 (Student’s t test). Differences between quiescent and 24 h or 24 h, 48 h and 72 h post-PH are indicated by ^†^p < 0.05, ^††^p < 0.01 and ^†††^p < 0.001 (Student’s t test).

In the early phases of liver regeneration after PH, the liver undergoes robust metabolic adaptations to support proliferation, remodeling and differentiation, whilst maintaining essential liver functions. Several studies had previously reported on the importance of various cytokines in the early phases of liver regeneration (i.e. first few hours) after PH^1^. In fact, it has been described that initiation of liver regeneration is defective in OPN deficient mice^18^. We recently reported that OPN regulates PC and cholesterol metabolism cross-talk in the quiescent mouse liver and that OPN deficient mice exhibit decreased *de novo* FA and TG synthesis in hepatocytes while liver TG content maintains unaltered^17^. Thus, we first evaluated if OPN plays a role in modulating the liver lipidome during regeneration after 70% PH. The results show that serum OPN levels increase by nearly 2-fold 24 h after PH in WT mice and remain elevated 48 and 72 h after PH (Fig. 1A). Compared with WT mice, body weight loss was significantly lower in OPN-KO mice 24 and 48 hours after PH (Fig. 1B). These findings suggest that OPN regulates whole body metabolism during liver regeneration.

FAs are essential constituents of glycerolipids, and have different metabolic fates depending on their chain length and degree of saturation^19^. Therefore, to ascertain whether OPN deficiency could be associated with changes in the liver lipidome during regeneration, TG and PC species from quiescent livers and regenerating livers 24 and 48 h post-PH were analyzed (Fig. 1C). We found that 52% of the analyzed TG species were increased in quiescent OPN-KO mouse livers when compared with livers from WT mice (Fig. 1C; Supplemental Table 1), but these differences abated with liver regeneration (Fig. 1C; Supplemental Table 1). Changes in PC species similarly occurred after PH in OPN-KO mice. Specifically, 35% of the analyzed PC species were decreased in quiescent OPN-KO mice but differences between OPN-KO and WT mice minimized 24 h after PH, (Fig. 1C; Supplemental Table 1) and were maintained 48 h post -PH (Fig. 1C; Supplemental Table 1). In OPN-KO mice, the fluctuations in several TG species along regeneration (Fig. 1C; Supplemental Table 1), which show enrichment is some TG species but not in others, were not associated with changes in concentration of liver TG (Fig. 1D) or accumulation of lipid droplets (Fig. 1D), while those of PC species were linked to decrease PC concentration in quiescent and regenerative liver (Fig. 1D). PCs can be catabolized to fatty acids and diglycerides through the action of different phospholipases being a source of TGs^7,20^. Here we observed that activation of phospholipase D (PLD) (Fig. 1E) or of calcium dependent phospholipase A2 (PLA2) (Fig. 1F) remained unaltered in the quiescent OPN-KO liver. Besides, the results showed that in liver of OPN deficient mice, there is no change in the LysoPC to PC ratio (Fig. 1E), which is an indirect indicator of PLA2 activity.

**Figure 2:**
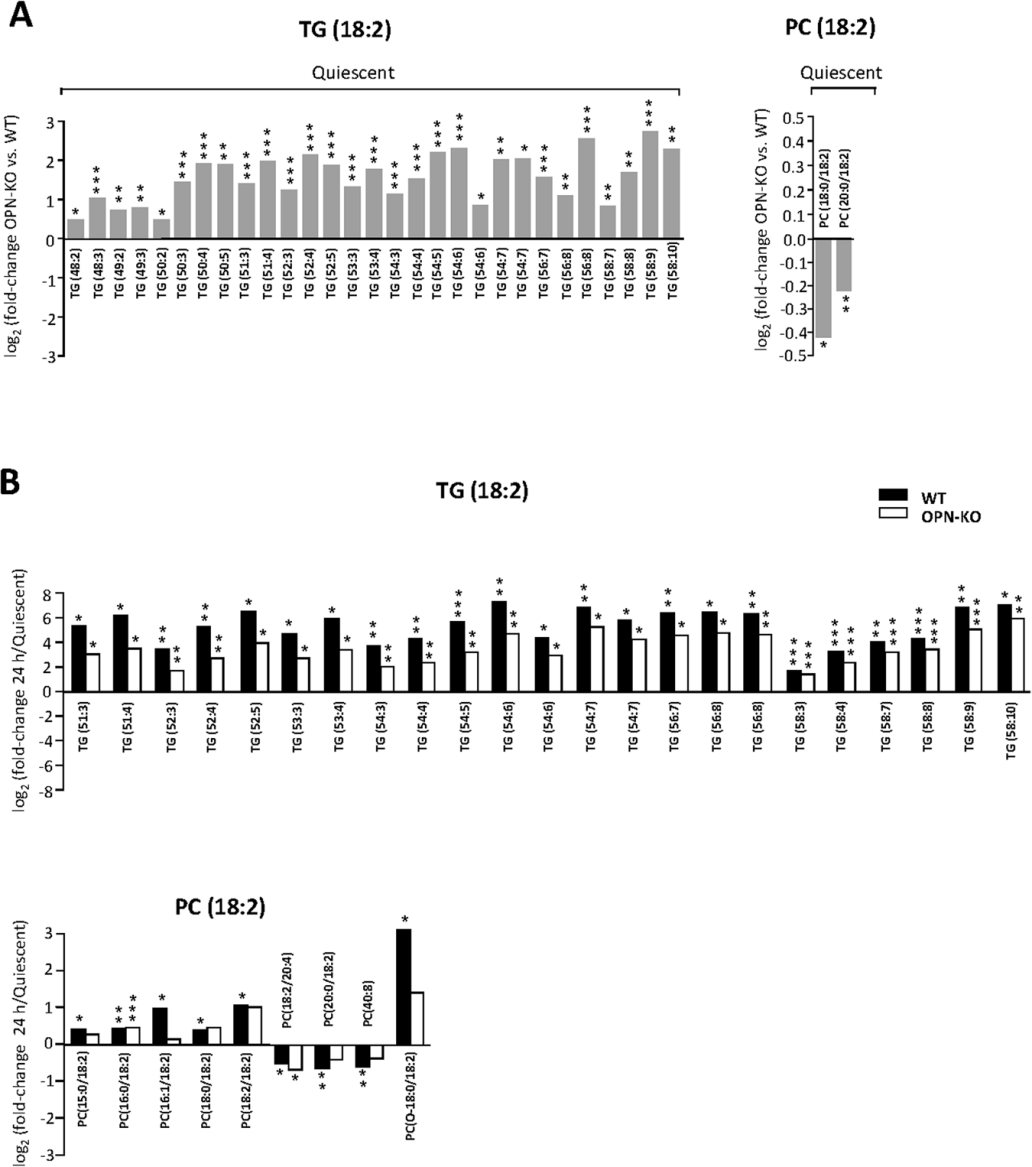
Liver triacylglycerol is enriched in the dietary linoleic acid (18:2) in OPN-KO mice. Osteopontin knockout (OPN-KO) mice and their controls (WT) were subjected to partial hepatectomy (PH), and liver was collected before and 24 h post-PH. Triacylglycerol (TG) and phosphatidylcholine (PC) species containing the essential linoleic acid (18:2) were analyzed (**A**) comparing differences in the quiescent liver species between both genotypes and (**B**) differences during the first 24 h of liver regeneration in each genotype. The results are represented as log_2_ of the fold-change. Values are mean of the fold -change from 5 animals per group and significant differences are denoted by *p < 0.05, **p < 0.01 and ***p < 0.001 (Student’s t test).

Considering that PC and TG *de novo* synthesis are reduced in OPN-KO mice hepatocytes while liver TG concentration remains unaltered^17^, to know the contribution of dietary FAs we next analyzed if TG and PC species containing the essential linoleic acid (18:2) were altered. We found that most of the TG species that were increased in OPN-KO livers were enriched in linoleic acid (18:2); these FAs cannot be synthesized by the cell and must be acquired from the diet (Fig. 2A; Supplemental Table 1). We also observed that PC species containing the linoleic acid (18:2) were reduced in the quiescent OPN-KO livers when compared with WT livers (Fig. 2A; Supplemental Table 1). In aggregate, these results suggest that the decreased *de novo* TG synthesis detected in OPN-KO mice liver is compensated by increased dietary FA uptake. This compensation however, does not occur with PC loss.

Since the big changes in liver lipid content during regeneration occur mainly 24 hours after PH, we also evaluated if the increased TG storage was associated with the enrichment in linoleic acid. The results showed that increase in linoleic acid-containing TG species was significantly more marked in WT compared with OPN-KO mice liver (Fig. 2B; Supplemental Table 1). Changes in linoleic-acid containing PC species however, were less consistent than the former (i.e. some containing the linoleic acid increased while other decreased 24 h after PH) (Fig. 2B; Supplemental Table 1).

### Liver lipid oxidation rate is increased in OPN-KO mice 24 h post-PH

**Figure 3:**
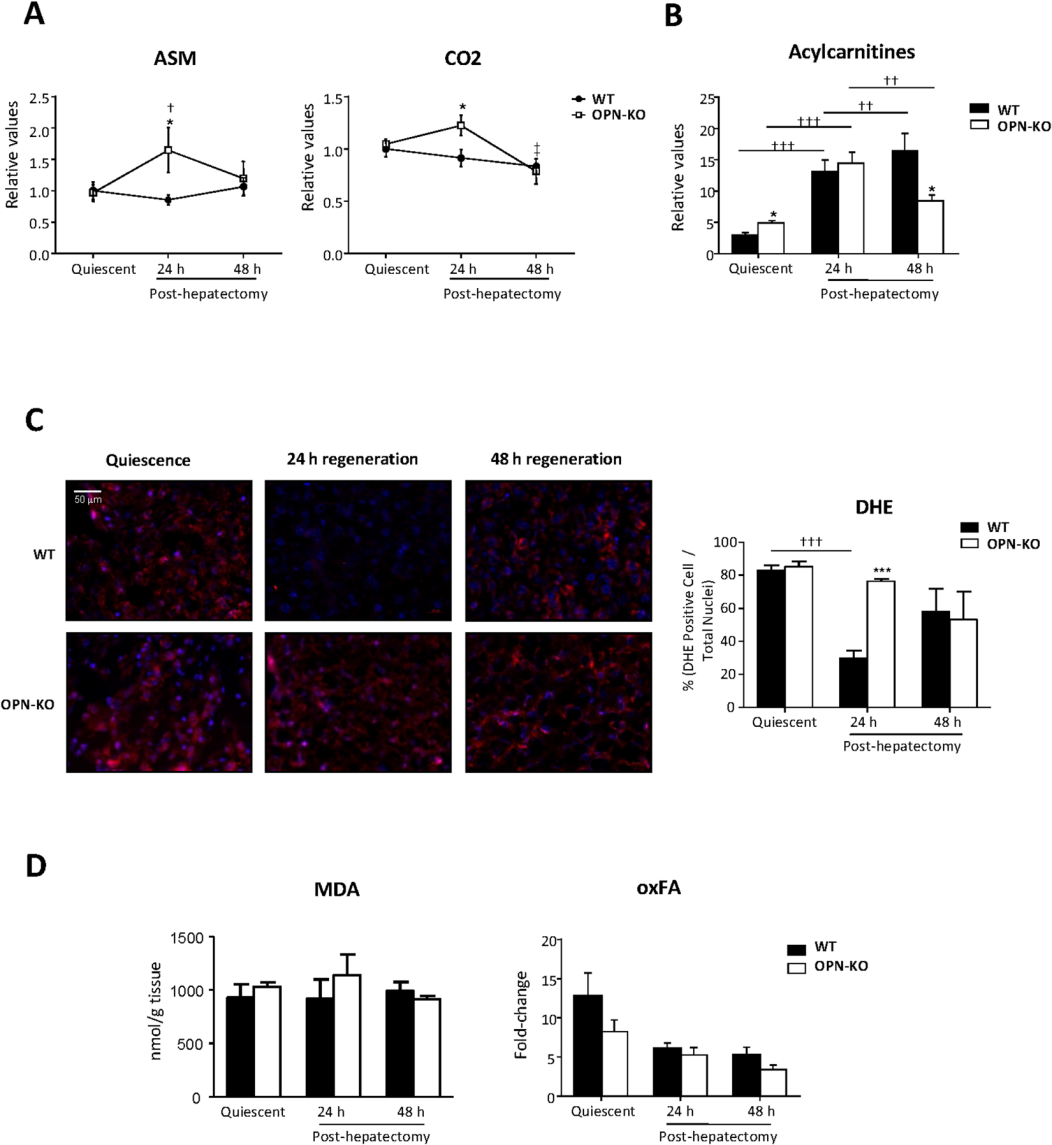
Lipid oxidation is higher in OPN-KO mice than in WT mice 24 h after partial hepatectomy. Osteopontin knockout (OPN-KO) mice and their controls (WT) were subjected to partial hepatectomy (PH), and livers were collected before and 24 h and 48 h post-PH. (**A**) Fatty acid oxidation assay was performed and the released radioactive acid soluble metabolites (ASM) and CO_2_ were quantified. (**B**) Total acylcarnitine content was measured. (**C**) Representative liver sections of DHE staining and quantification. (**D**) Malondialdehyde (MDA) levels in liver samples represented in nmol/gram of tissue (left) and the content of oxidized fatty acids (FA) (right). Values are mean ± SEM from 4–5 animals per group. Significant differences between OPN-KO and the corresponding WTs are denoted by *p < 0.05 (Student’s t test); Significant differences along regeneration are indicated by ^†^p < 0.05, ^††^p < 0.01 and ^†††^p < 0.01 (Student’s t test).

Transient liver TG accumulation during PH has been correlated with changes in lipid oxidation^21,22^. Herein, we assessed liver lipid oxidation rate by measuring the rate of conversion of ^14^C- palmitate into acid-soluble metabolites (ASM) or CO_2_ (Fig. 3A). 24 h post-PH, incomplete (ASM) and complete (CO_2_) lipid oxidation was increased in OPN -KO mice when compared to WT mice (Fig. 3A). By contrast, no differences were seen in the quiescent liver or in the regenerating liver 48 h post-PH.

For mitochondrial lipid oxidation to occur, FAs need to be converted into acylcarnitine by CPT-1 before being transported from the cytosol. Importantly, altered acylcarnitines content has been associated with changes in lipid oxidation^23^. In this study, we observed that acylcarnitine levels were higher in livers of OPN-KO mice under basal (quiescent) conditions. Although acylcarnitine levels were increased in both OPN-KO and WT mice 24 after PH, the increase was more marked in WT mice (Fig. 3B), in which beta oxidation was not increased (Fig. 3A). The increase in acylcarnitine content was 4.5 fold for the WT mice while it was 2.9 fold for OPN-KO mice. 48 h post-PH, acylcarnitines were even higher in WT mice while decreased in the KO mice (Fig. 3B).

Next, we found that 24 h post-PH, the increased lipid oxidation was associated with increased percentage of cells positive for dihydroethidium (DHE) (Fig. 3C), which have been used extensively to evaluate reactive oxygen species (ROS) production while no differences were observed in malondialdehyde (MDA) (Fig. 3D), which is a natural bi-product of lipid peroxidation, or in oxidated fatty acids (FA) (Fig. 3D, Supplemental Fig. 1B).

### Lysosomal TG hydrolase activity is increased in livers from OPN-KO mice while circulating TG and PC into lipoproteins are unchanged

**Figure 4:**
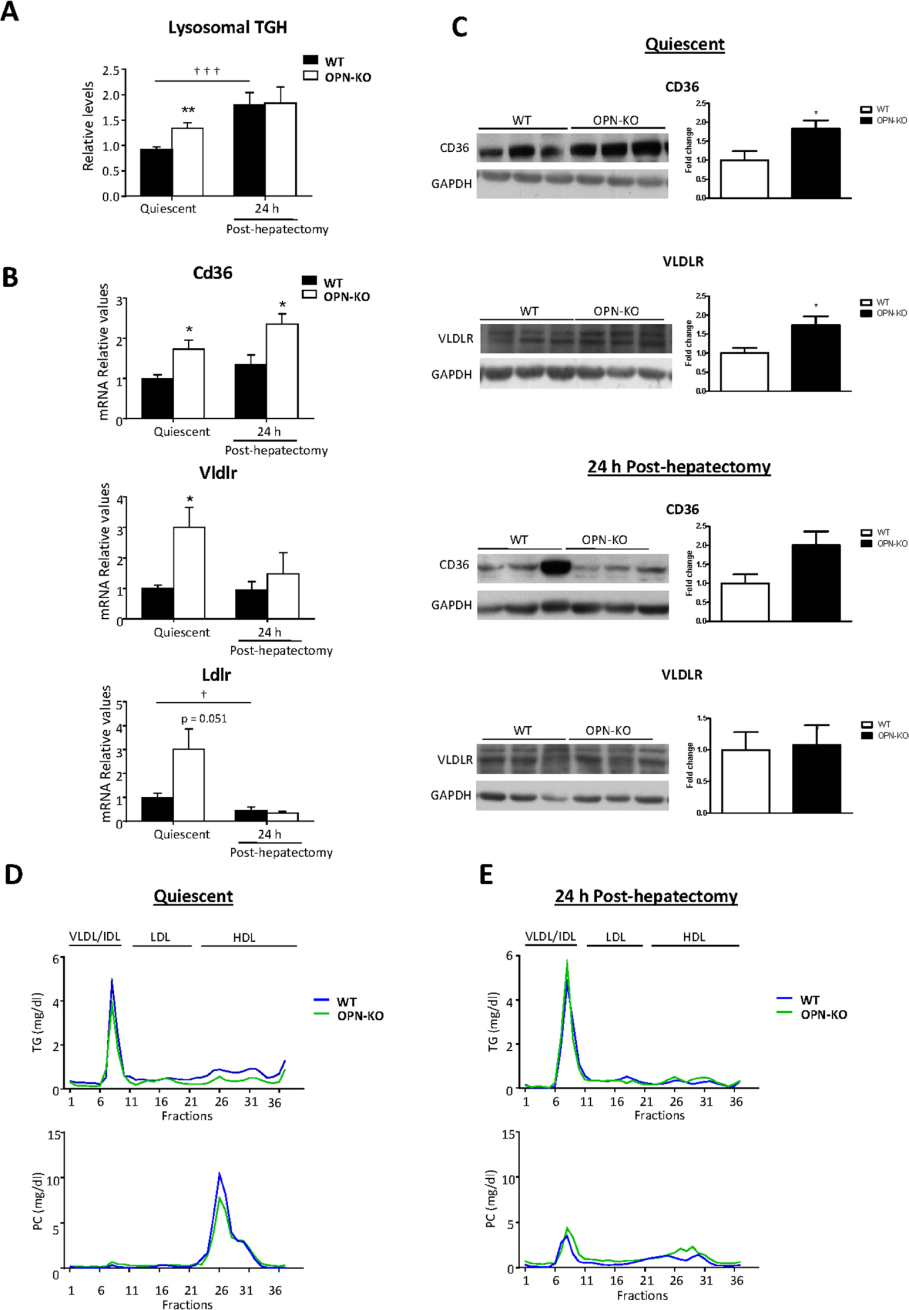
Increased liver lysosomal triacylglycerol hydrolase activity in OPN-KO mice is not linked with changes in circulating lipoprotein triacylglycerol and phosphatidylcholine distribution. Osteopontin knockout (OPN-KO) mice and their controls (WT) were subjected to partial hepatectomy (PH) and livers and serum from quiescent and 24 h post-hepatectomy were collected. (**A**) Lysosomal triacylglycerol hydrolase (TGH) activity was determined using a radiometric assay. (**B**) Hepatic mRNA levels of Cd36, very-low-density lipoprotein receptor (Vldlr) and low-density lipoprotein (Ldlr) genes were measured by rt-qPCR. (C) CD36 and very-low-density lipoprotein receptor (VLDLR) protein levels were assessed by inmmunoblotting. In all the immunoblots glyceraldehyde-3-phosphate dehydrogenase (GAPDH) was used as a loading control. Serum lipoproteins were separated into 37 subclasses (represented as fraction numbers) (**D**) in quiescence and (**E**) 24 h post-hepatectomy. Triacylglycerol (TG) and phosphatidylcholine (PC) concentrations were determined in the fractionated lipoproteins: very-low/intermediate-density lipoproteins (VLDL/IDL; fractions 1 to 11), low-density lipoproteins (LDL; fractions 12 to 21) and high-density lipoproteins (HDL; fractions 22 to 37). Values are mean ± SEM of 4–8 animals per group. Statistical differences between OPN-KO and the corresponding WT mice are denoted by *p < 0.05 and **p < 0.01 (Student’s t test) and differences along regeneration are indicated by ^†^p < 0.05 and ^†††^p < 0.001 (Student’s t test).

Given that liver lipid beta oxidation was higher (Fig. 3A) 24 post-PH in OPN-KO mice as compared to WT mice while there were no changes in liver TG concentration, we next evaluated if increased liver lipoprotein uptake could be involved in providing the energy source required. The results showed that basal lysosomal TG hydrolase (TGH) activity was higher in the OPN-KO liver (Fig. 4A) and that TGH activity increased 24 h post-PH in WT mice (Fig. 4A). In the quiescent liver, Cd36 and very-low-density lipoprotein receptor (Vldlr) mRNA (Fig. 4B) and protein levels (Fig. 4C) were higher in OPN-KO mice than in WT mice. There was also and increase in low-density lipoprotein receptor (Ldlr) mRNA levels. After PH, Cd36 mRNA remained significantly higher in OPN-KO livers compared with WT livers (Fig. 4B) while no statistically significant difference were found between groups in Vldlr, Ldlr gene expression (Fig. 4B) or in CD36 and VLDLR protein levels (Fig. 4C).

Serum lipoproteins have been considered a source of lipids for the transient regeneration-associated steatosis in the liver^24^. Thus, we also analyzed whether these changes in lysosomal TGH activity and expression of lipoprotein receptors could be linked with changes in serum TG and PC distribution into lipoproteins. Lipid distribution into serum lipoproteins showed that OPN deficiency did not alter TG or PC distribution into serum VLDL/ intermediate-density lipoprotein (IDL), LDL or high-density lipoprotein (HDL) in quiescent livers (Fig. 4D). The TG distribution into serum lipoproteins was also comparable in both WT and OPN-KO mice 24 h post-PH (Fig. 4E). PC distribution on the other hand, was altered 24 h after PH but changes were similar between OPN-KO and WT mice (Fig. 4D,E).

### Atorvastatin induced changes in the lipidome 24 h after PH and promoted early liver regeneration in OPN-KO mice

**Figure 5:**
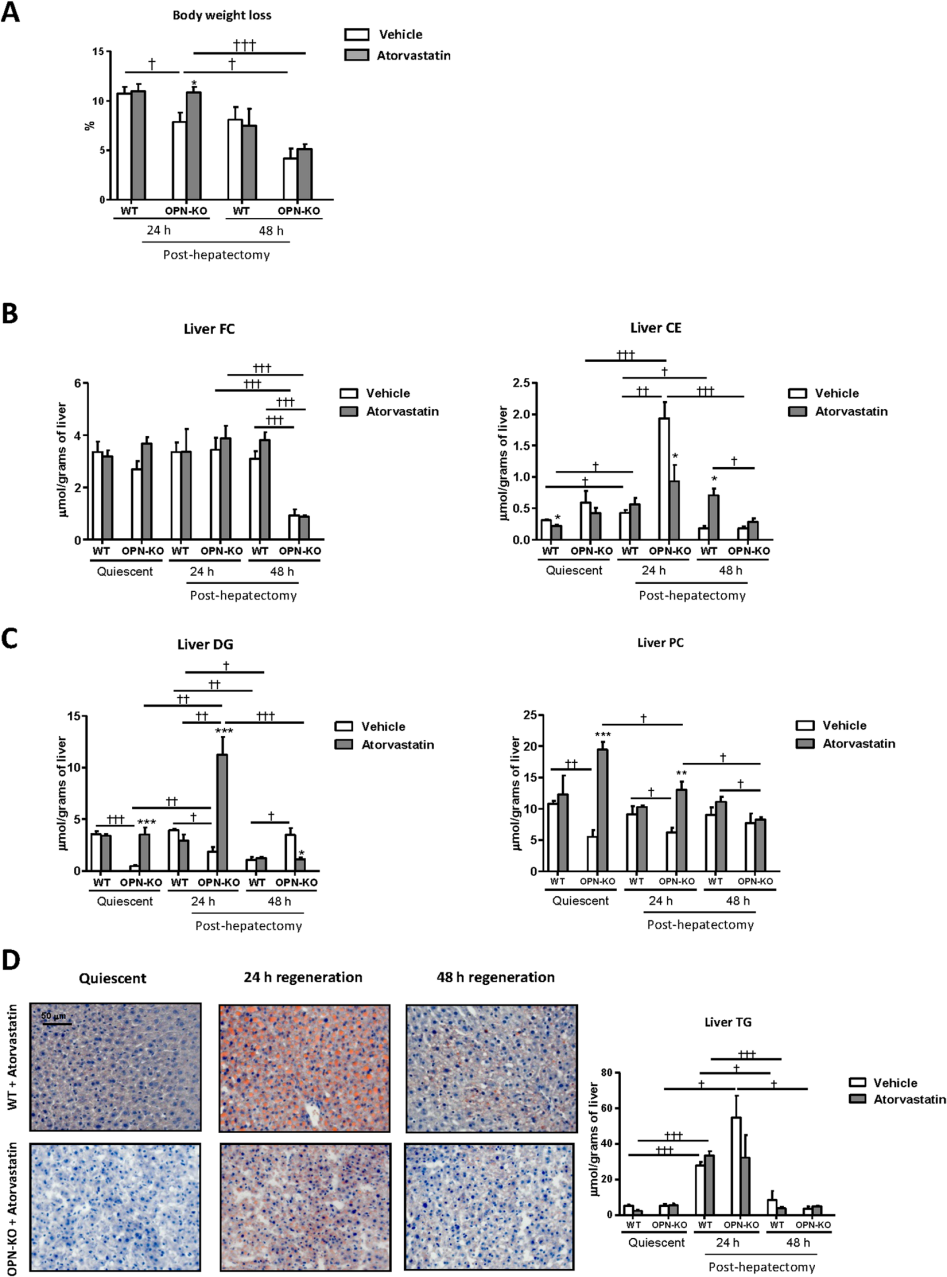
In OPN-KO mice atorvastatin treatment induces changes in liver lipidome 24 h after partial hepatectomy. Osteopontin knockout (OPN-KO) mice, OPN-KO mice treated with atorvastatin (100 mg/kg), and their respective controls treated or untreated with atorvastatin (WT) were subjected to partial hepatectomy (PH) and livers were collected before and 24 h and 48 h post-hepatectomy. (**A**) Percentage of body weight loss. (**B**) Liver free cholesterol (FC), cholesteryl ester (CE). (**C**) diacylglycerol (DG) and phosphatidylcholine (PC) content are represented in nmol/gram of liver. (**D**) Representative liver sections stained with Sudan III (left) and liver triacylglycerol (TG) content (right) is represented in nmol/gram of liver. Values are mean ± SEM of 4–8 animals per group. Statistical differences between treated or untreated mice within one genotype are denoted by *p < 0.05 (Student’s t test) and differences along regeneration and between genotype are indicated by ^†^p < 0.05, ^††^p < 0.01 and ^†††^p < 0.001 (Student’s t test).

The greatest increase in serum OPN levels occurred during the first 24 h after PH because no changes were observed between 24 and 48 h (Fig. 1A). This increase in serum OPN coincided with the peak of body weight loss (Fig. 1B). Recently, we demonstrated that *in vivo* inhibition of cholesterogenesis in OPN-KO mice livers with atorvastatin restores PC content^17^. In this study, we further observed that OPN modulates liver lipid metabolism (and liver lipidome) during regeneration after PH (Fig. 1B,C). Therefore, we evaluated if atorvastatin could remodel liver lipid metabolism in OPN-KO mice and analyzed the impact of atorvastatin on liver regeneration after PH. We found that in OPN-KO mice treatment with atorvastatin resulted in greater body weight loss 24 h after PH (Fig. 5A). This effect was not observed in WT mice (Fig. 5A). Atorvastatin treatment did not alter liver free cholesterol (FC) levels in WT or KO mice (Fig. 5B). Levels of cholesteryl ester (CE) (i.e. a reflection of cholesterol storage) however, were repressed with atorvastatin 24 h after PH just in OPN-KO mice (Fig. 5B). Atorvastatin also increased liver DG and PC content 24 h after PH in OPN-KO mice inducing no effect in WT mice (Fig. 5C), as we had observed in the quiescent livers^17^ (Fig. 5C). Atorvastatin treatment did not induce changes in the accumulation of lipid droplets or in the liver TG content 24 h after PH in the OPN-KO or WT mice (Fig. 5D). Atorvastatin treatment decreased liver DG content in OPN-KO mice 48 h after PH (Fig. 5C) while it did not induce changes in body weight loss or concentration in other lipids (Fig. 5A,B,D). Since we have observed that in OPN-KO mice the increased lipid oxidation was associated with increased DHE, here we wanted to know if this maintained increased after the atorvastatin treatment, that restores the lipidome. The results showed that differences between WT and OPN-KO in DHE disappeared after atorvastatin treatment (Supplemental Fig. 1).

**Figure 6:**
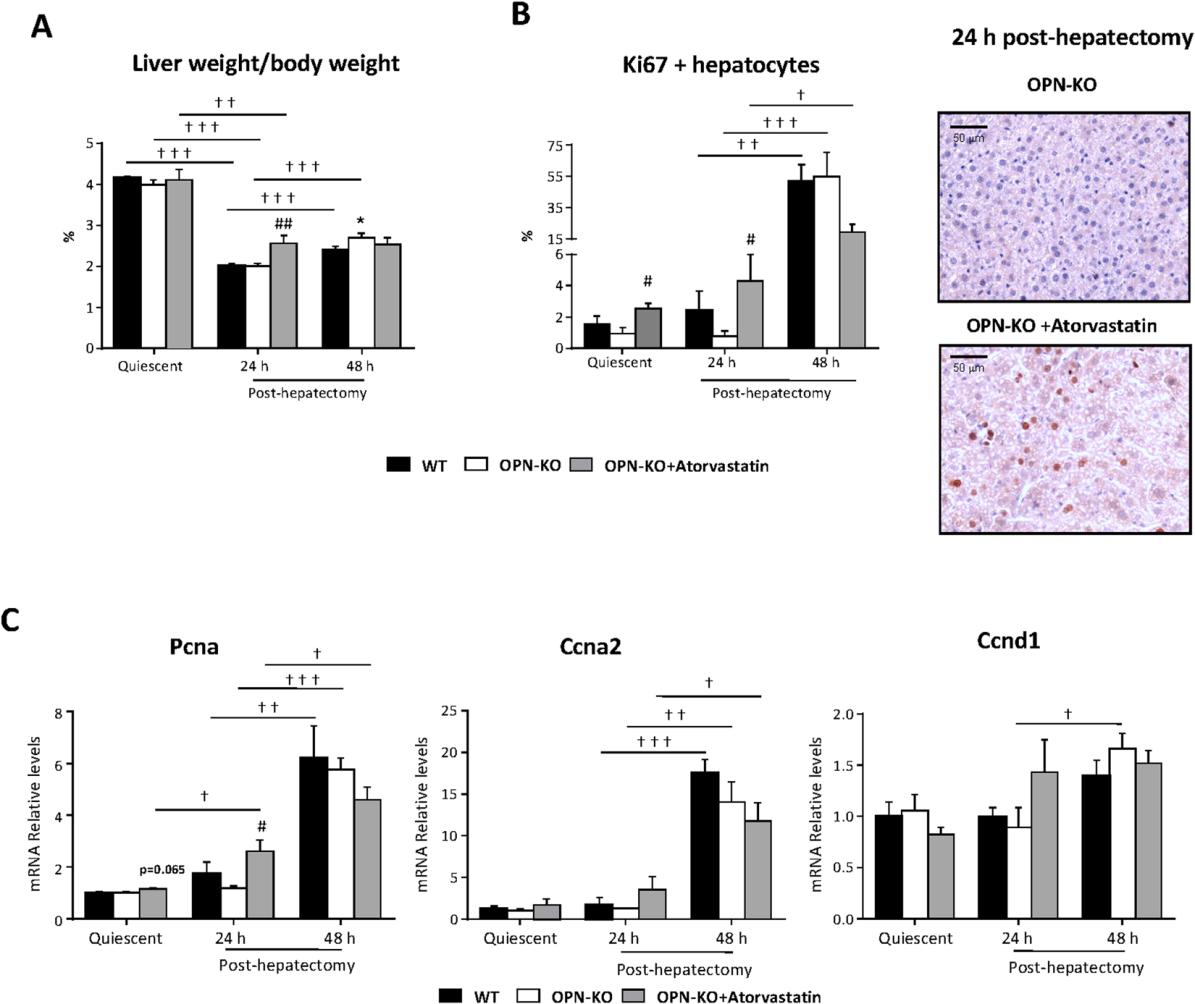
Atorvastatin improves regeneration 24 h after partial hepatectomy in OPN-KO mice. Wild type (WT), osteopontin knockout (OPN-KO) and OPN-KO treated with atorvastatin (100 mg/kg) mice were subjected to partial hepatectomy (PH) and livers from quiescent, 24 h and 48 h post-hepatectomy were collected. (**A**) The percentage of body/liver weight was calculated. (**B**) Ki67 immunostaining was performed. (**C**) Hepatic mRNA levels of genes implicated in cell cycle progression were measured. Values are mean ± SEM from 5–10 animals per group. Significant differences between OPN-KO and WT mice are denoted by *p < 0.05 (Student’s t test). Differences between OPN-KO and OPN-KO treated with atorvastatin are indicated by #p < 0.05 and ##p < 0.01. Differences along regenration are indicated by ^†^p < 0.05, ^††^p < 0.01 and ^†††^p < 0.001 (Student’s t test).

Administration of atorvastatin to OPN-KO mice led to an increase in the liver to body weight ratio (Fig. 6A), in the percentage of Ki67 positive hepatocytes (Fig. 6B) and in the expression of the proliferating cell nuclear antigen (Pcna) (Fig. 6C) 24 h after PH as compared to non-treated OPN-KO mice. Atorvastatin did not alter mRNA levels of cyclin A2 (Ccna2) and cyclin D1 (Ccnd1) (Fig. 6C) and did not affect regeneration 48 h after PH (Fig. 6A–C, Supplemental Fig. 2). No changes in any of these parameters were observed in OPN-KO mice when compared with the WT mice (Fig. 6A–C). Atorvastatin treatment did not alter the liver to body weight ratio (Supplemental Fig. 3A) or the percentage of Ki67 positive hepatocytes (Supplemental Fig. 3B) in WT mice. In aggregate, these results suggest that atorvastatin induces liver regeneration in OPN-KO mice only 24 h post-PH, when it induces a new lipidome profile.

## Discussion

OPN is a multifunctional cytokine involved in different liver disorders related with activation of a regenerative response, such as obesity related steatosis^25^, non-alcoholic steatohepatitis^15^, fibrosis^15^, or hepatocellular carcinoma^26^. Different studies have also shown that OPN is involved in liver regeneration after PH^16,18^. Wen *et al*.^18^ demonstrated that in mice OPN is required for hepatocyte proliferation during the priming phase; Wang *et al*.^16^ showed that in rats OPN overexpression aggravated hepatic necrosis and leukocyte infiltration while OPN silencing inhibited liver regeneration rate in later stages than in mice.

Liver regeneration is an important topic of study due to its implication in liver diseases and its importance in auxiliary (split) liver transplantations^27,28^. For a successful initiation and completion of liver regeneration the remaining cells within the liver need to acquire sufficient energy and precursors to support the metabolic demands of rapid proliferation, which involve an immense metabolic remodeling^1^. We previously showed that OPN regulates *de novo* lipogenesis in hepatocytes and glycerolipid metabolism^17^. So, there is a need to understand how OPN affects lipid metabolism during regeneration. In this study, we show that there is a substantial increase in the levels of circulating OPN during the first 24 h after PH, occurring at a time when hepatocytes accumulate a significant amount of lipids (mainly TGs), in droplets, in the widely recognized transient regeneration-associated liver steatosis^9,29^. Evidences suggest that the transient steatosis is likely related to the increase lipogenic activity and the altered lipid fluxes into and out of the hepatocyte^9,21,30^.

Previously, we also demonstrated that *de novo* TG synthesis is decreased while the liver TG content maintains unaltered in OPN-KO hepatocytes^17^. Here, we further show that enrichment of liver TGs in the most abundant dietary essential FA, the linoleic acid (18:2), occurs in OPN-KO mice. This enrichment is associated with an increase in the lysosomal TGH activity, involved in the internalized lipoprotein lipid hydrolysis. All together, these results show that the decreased *de novo* TG synthesis is compensated with dietary FA in OPN-KO mice livers. During the first 24 h period after PH, the enrichment of TGs in linoleic acid was higher in the WT than in OPN-KO mice, in which beta oxidation was increased. All together, suggesting an increased catabolism of dietary fatty acids.

Disruptions in hepatic adipogenesis have been associated with impaired liver regeneration in mice^31^. Our findings demonstrated that in OPN-KO female mice liver, in which the source of TGs is different, regeneration was not impaired 24 or 48 h after PH. It has also been described that during the first hours of the G1 phase there is an increase in the TG hydrolysis that releases FAs for mitochondrial beta oxidation^32^. Here we observed that in OPN-KO mice there is an increase in beta oxidation 24 h after PH, which prevents the accumulation of acylcarnitines observed in quiescent livers and will induce the decrease in acylcarnitines 48 h after PH. This increase in beta oxidation is coupled with the increase in positive cells for DHE, a marker of ROS. It has been described that the hypoglycaemia that follows PH induces systemic lipolysis which supplies the required FA^8^. The loss of body weight that follows the PH is less marked in OPN-KO mice than in WT mice, suggesting that lipolysis of adipose tissue is not the source of FA for the increased beta oxidation. These results are in concordance with the fact that in mice, OPN deletion, prevents the development of obesity and hepatosteatosis via impaired adipose tissue functionality^25^. Our results suggest that dietary FA will provide the required TG and FA for lipid accumulation and beta oxidation in OPN-KO mice.

The synthesis of PC, the major phospholipid in cellular membranes, is coordinated with the cell cycle activation^2^. However, lack of CTP:phosphocholine cytidyltransferase in mice, the rate limiting enzyme responsible for 70% of liver PC^5^, does not attenuate liver regeneration after PH^6^. Our results show that liver regeneration is not impaired in OPN-KO mice even when PC content is decreased 24 h post PH. We also found that the decreased *de novo* PC synthesis previously observed is associated reduced liver PC content, but unlike TGs, PC content is not compensated with the dietary essential FA linoleic. Importantly, treatment with atorvastatin, which inhibits cholesterogenesis, induces a new lipidome landscape where PC and DG content increases 24 h after PH. This new metabolic rewiring was associated with improved liver regeneration 24 h after PH, and is consistent with our previous reports of increased *de novo* cholesterogenesis in OPN-KO mice.

This study reinforces the role of OPN as a metabolic driver during liver regeneration. However, in this mice model, non-obese with healthy liver, the lack of OPN results in a metabolic remodeling ensuring the success in regeneration 24 and 48 h after partial hepatectomy. OPN is increased in liver diseases and the knockdown of OPN has been proposed as a therapeutical approach. However, through this study we are not able to know if the lack of OPN will alter liver regeneration in a model of obesity with non-alcoholic fatty liver disease. Liver transplants are increasing due to metabolic disease. Thus, this should be investigated in the future.

In conclusion, the results here support the role of OPN as a liver metabolic driver. OPN regulates multiple metabolic pathways involved in liver regeneration. Treatment with atorvastatin, an inhibitor of *de novo* cholesterogenesis, provides a new metabolic scenario linked to improvement of early regeneration in OPN-KO mice.

## Methods

### Animals

10–12-week-old female OPN-KO mice and their WT littermates were provided by Jackson´s Laboratories. They were maintained on a rodent chow diet (Teklad Global 18% Protein Rodent Diet 2018S; Harlan Laboratories INC., USA) and were housed in a temperature-controlled room with a 12 hour-light/ dark cycle. Animal procedures were approved by the Ethics Committee for Animal Welfare of the University of the Basque Country UPV/EHU and were conducted in conformity with the EU Directives for animal experimentation.

### Partial hepatectomy

Mice were subjected to 70% PH, as previously described^33^ under general anesthesia with inhaled isoflurane. Animals were sacrificed at different times (24 h, 48 h and 72 h) after surgery.

### Atorvastatin treatment

Intragastric atorvastatin (100 mg/kg) was provided to a group of OPN deficient animals during 2 weeks in alternate days. 24 h hours after the last dose the mice were subjected to 70% PH and were sacrificed at different times (24 h and 48 h) after surgery.

### Liver lipidomic analysis

Liver lipid profiles were analyzed in OWL as described previously^34^. Briefly, two separate UPLC-time-of- flight (TOF)-mass spectrometry (MS)-based platforms analyzing methanol and chloroform/methanol liver extracts were combined. Identified ion features in the methanol extract platform included acylcarnitines, monoacylglycerophospholipids (LPC) and oxidized FAs. The chloroform/methanol extract platform provided coverage over glycerolipids. Lipid nomenclature follows the LIPID MAPS convention (www. lipidmaps.org)

### FA oxidation

Beta oxidation was assessed as described before^35,36^. Fresh liver pieces were homogenated in a Potter homogenizer (5 strokes) in cold buffer (25 mM Tris-HCl, 500 nM sucrose, 1 mM EDTA-Na_2_ pH 7,4) and sonicated for 10 s. Then, the homogenates were centrifuged at 500 × g for 10 min at 4 °C. Approximately 500 µg of protein from the homogenates supernatant was used for the assay in a volume of 200 µl. The reaction started by adding 400 µl of assay mixture containing 0.5 µCi/ml [1-^14^C] palmitic acid to the samples and was incubated for 1 h at 37 °C in eppendorf tubes with a Whatman paper circle in the cap. The reaction was stopped by adding 300 µl of 3 M perchloric acid and 1 M NaOH was added to impregnate the whatman cap.

After 2 h the Whatman caps were retired and the radioactivity associated was measured in a scillation counter. The eppendorf tubes were centrifugated at 21,000 × g 10 min at 4 °C. 400 µl from the supernatant were collected and the radioactivity was counted in a scintillation counter. The supernatant contained the acid soluble metabolites (ASM) and the Whatman caps captured the released CO_2_.

### Analysis of liver lipid concentration

After homogenization of liver tissue, lipids were extracted as described before^37^. PC, FC, CE and DG were quantified as described previously^38^ and TGs were quantified using a commercially available kit (A. Menarini Diagnostics, Italy).

### TG and PC distribution in serum lipoprotein subclasses

Serum lipoproteins were separated using an AKTA-fast-protein liquid chromatography using a Superose 6 10/300 GL column (GE Healthcare Europe GmbH, Germany) as detailed^39^. After equilibration, 200 µl of serum were applied and fractions were collected. Commercially available kits were used to measure TG (A. Menarini Diagnostics, Spain) and PC (Spinreact, Spain) in each fraction.

### Lipid peroxidation Assay kit

Malondialdehyde (MDA) content is generally used as marker for lipid peroxidation. MDA content in liver samples was quantified by using a commercially available kit from Sigma-Aldrich (St. QuentilFallavier, France).

### Determination of ROS in liver tissue sections

Samples were sectioned in a criostat (8 μms), and incubated with MnTBAP 150 μM at RT during 1 h. The samples were then incubated with DHE 5 μM for 30 min at 37 °C^40^. Sections were mounted with mounting media containing DAPI.

### Osteopontin ELISA

During regeneration, OPN was quantified in the serum of WT mice using OPN Quantikine ELISA kit (R&D Systems) according to the manufacturer’s protocol.

### Histology

A piece of liver was formalin-fixed and 4 microm-thick sections were stained with hematoxylin and eosin (Neiker-Tecnalia, Spain) following standard methods.

For the histological evaluation of lipid storage in liver, Sudan Red staining was performed. Briefly, cut liver cryostat section of 8 microms were incubated with freshly-prepared Sudan III stain (Sigma-Aldrich) and contrasted with Mayers hematoxylin. Stained area percentage of each sample were calculated using FRIDA software (FRamework for Image Dataset Analysis) http://bui3.win.ad.jhu.edu/frida/.

### Inmunoassays

To quantify the regeneration rate, Ki67 immunostaining of formaline fixed liver sections was performed^24^ by incubation with anti-Ki67 primary antibody (1:1500) (Novocastra Reagents, UK) at 4 °C overnight.

For the immunoblots, samples were subjected to sodium dodecyl sulfate-polyacrylamide gel electrophoresis (SDS-PAGE) and proteins were transferred to Immobilon-P membranes. Western blotting was performed using different primary antibodies: Phosphorylated (Thr-147) phospholipase D1 (P-PLD1) and total PLD1 from Cell Signaling Technology, calcium free posphorylated (Ser-505) phospholipase A2 (P-PLA2), total PLA2 and glyceraldehyde-3-phosphate dehydrogenase (GAPDH) from Abcam, CD36 from NOVUS and VLDLR from R&D Systems.

### Analysis of lysosomal TGH activity

Lysosomes enriched fractions were isolated from liver homogenates by differential centrifugation essentially as described before^41^. TGH enzymatic activities were measured using radiometric assays as previously described^42,43^.

### RNA extraction, cDNA synthesis and qPCR

Total RNA was extracted using Trizol Reagent (Invitrogen, Spain) and cDNAs were obtained by retrotranscription (SuperScript III RT, Invitrogen, USA) following the manufacturers’ instructions.

cDNA was used for specific target amplification using the Qiagen Multiplex PCR Master, after 14 cycles of amplification (95 °C 15 min, 14 cycles 95 °C for 15 sec and 60 °C for 4 min), amplified cDNA was treated with Exo I following Fluidigm protocol instructions, diluted 1:5 with low EDTA TE buffer and loaded onto 48.48 or 96.96 Dynamic Array IFC. SsoFast^TM^ EvaGreen® Supermix with Low ROX (Bio-Rad Laboratories, USA) is used for amplification. The cycling program consisted of 1 min at 95 °C, 35 cycles of 95 °C for 5 sec and 60 °C for 20 secs, followed by a melting curve.

The expression of the selected genes was measured by RT-qPCR using the BioMarkTM HD system in combination with Dynamic Array Integrated Fluidic Circuits (IFC) (Fluidigm Corporation, USA) following Fluidigm’s Fast Gene Expression Analysis using EvaGreen on the BioMark HD System version D1 protocol. Finally, the stability of candidate reference genes was analyzed with NormFinder algorithms and all the data analysis was performed using GenEx software. The oligonucleotides and sequences used for quantitative PCR analysis were the following ones: Cd36 (Fw: CCCTCCAGAATCCAGACAAC; Rv: CACAGGCTTTCCTTCTTTGC), Vldlr (Fw: TGACGCAGACTGTTCAGACC; Rv: GCCGTGGATACAGCTACCAT) and Ldlr (Fw: AGGCTGTGGGCTCCATAGG; Rv: TGCGGTCCAGGGTCATCT). All reactions were performed in duplicate, and expression levels were normalized to the average level of Gapdh (Fw: TATGACTCCACTCACGGCAAATT; Rv: TCGCTCCTGGAAGATGGTGAT), Ppia (Fw: CCAAGACTGAGTGGCTGGATG; Rv: GCTCCATGGCTTCCACAATG), and Actb (Fw: ATCGCTGACAGGATGCAGAAG; Rv: TCAGGAGGAGCAATGATCTTGA) in each sample.

### Total protein measurements

Protein concentration was measured using commercially available Bicinchoninic Acid Reagent (Thermo Fisher Scientific Inc).

### Statistical analysis

Data are represented as mean ± SEM. Differences between groups were tested using the Student’s t test. Significance was defined as p < 0.05. These analyses were performed using GraphPad Prism software.

## Electronic supplementary material

Supplementary Information

## Data Availability

The datasets generated during and/or analyzed during the current study are available from the corresponding author on reasonable request.
