# Supplementary material for: Atorvastatin provides a new lipidome improving early regeneration after partial hepatectomy in osteopontin deficient mice
Source: Sci Rep. 2018 Oct 2;8:14626. doi: 10.1038/s41598-018-32919-9 (PMC6168585; doi:10.1038/s41598-018-32919-9)

# Atorvastatin provides a new lipidome improving early regeneration after partial hepatectomy in osteopontin deficient mice

# Maitane Nuñez-Garcia^1^, Beatriz Gomez-Santos^1^, Diego Saenz de Urturi^1^, Daniela Mestre^1,2^, Francisco Gonzalez-Romero^1^, Xabier Buque^1,2^, Virginia Gutiérrez-de Juan^3^, María Luz Martinez-Chantar^3^, Wing-kin Syn^4,5^, Olatz Fresnedo^1^ and Patricia Aspichueta^1,2*^

^1^Department of Physiology, Faculty of Medicine and Nursing, University of the Basque Country, UPV/EHU, Spain; ^2^Biocruces Health Research Institute, Spain; ^3^Liver disease Laboratory, Liver metabolism Laboratory, CIC bioGUNE, Centro de Investigación Biomédica en Red de Enfermedades Hepáticas y Digestivas (CIBERehd), Spain. ^4^Division of Gastroenterology and Hepatology, Medical University of South Carolina, Charleston, South Carolina, USA; ^5^Section of Gastroenterology, Ralph H Johnson Veteran Affairs Medical Center, Charleston, South Carolina, USA

*Corresponding author: Patricia Aspichueta, Department of Physiology, University of the Basque Country UPV/EHU, Faculty of Medicine and Nursing, Sarriena s/n, 48940 Leioa, Spain. Phone: +34 946012896; Fax: +34 946015662; e-mail: patricia.aspichueta@ehu.eus

Running title: Osteopontin: a liver metabolic regulator during regeneration

**Keywords**: Osteopontin, liver regeneration, metabolism, phosphatidylcholine, triacylglyceride, lipidome.

Running title: Osteopontin: a liver metabolic regulator during regeneration

**Supplemental Table 1. Lipidomic analysis of triacylglycerol (TG) and phosphatidylcholine (PC) species** in OPN-KO and WT mice liver in quiescence and 24 h and 48 h after partial hepatectomy (PH).

| **TRIACYLGLYCERIDE** | | | | | | | |
| --- | --- | --- | --- | --- | --- | --- | --- |
| ***Individual notation*** | ***Individual composition or probable ID*** | ***p-value*** | ***Fold change*** | ***p-value*** | ***Fold change*** | ***p-value*** | ***Fold change*** |
|  |  | ***OPN-KO vs WT Quiescent*** | | ***OPN-KO vs WT 24 h*** | | ***OPN-KO vs WT 48 h*** | |
| **TG(48:2)** | TG(14:0+18:1+16:1)+ TG(16:0+18:2+14:0) | **4.27E-02** | 1.40 | 3.96E-01 | 0.61 | 4.64E-01 | 1.98 |
| **TG(48:3)** | TG(18:1+18:2+12:0) | **1.89E-04** | 2.05 | 4.76E-01 | 0.60 | 3.38E-01 | 2.13 |
| **TG(49:2)** | TG(15:0+18:2+16:0)+ TG(18:1+16:1+15:0)+ TG(18:1+14:0+17:1) + TG(16:0+18:2+15:0) | **5.33E-03** | 1.66 | 3.61E-01 | 0.57 | 3.11E-01 | 1.58 |
| **TG(49:3)** | TG(17:1+16:1+16:1) + TG(18:2+15:0+16:1) | **2.02E-03** | 1.71 | 4.21E-01 | 0.58 | 2.59E-01 | 1.55 |
| **TG(50:2)** | TG(16:0+18:1+16:1) + TG(18:1+18:1+14:0) + TG(16:0+18:2+16:0) | **2.89E-02** | 1.38 | 3.72E-01 | 0.63 | 3.75E-01 | 1.90 |
| **TG(50:3)** | TG(16:1+18:1+16:1) + TG(16:0+18:2+16:1) | **7.15E-07** | 2.72 | 3.46E-01 | 0.59 | 3.80E-01 | 1.86 |
| **TG(50:4)** | TG(16:1+18:2+16:1) + TG(16:1+18:3+16:0) + TG(18:1+18:3+14:0) + TG(14:0+18.4+18:0) | **3.15E-07** | 3.77 | 3.86E-01 | 0.55 | 3.77E-01 | 2.25 |
| **TG(50:5)** | TG(18:3+18:2+14:0) | **6.46E-03** | 3.67 | 6.36E-01 | 0.74 | 4.07E-01 | 2.32 |
| **TG(51:2)** | TG(16:0+17:1+18:1) | **7.75E-05** | 1.95 | 2.57E-01 | 0.56 | 3.61E-01 | 1.47 |
| **TG(51:3)** | TG(16:0+17:1+18:2) + TG(18:2+18:1+15:0) | **1.15E-04** | 2.62 | 2.55E-01 | 0.54 | 4.98E-01 | 1.46 |
| **TG(51:4)** | TG(18:2+18:2+15:0) + TG(17:1+18:2+16:1) + TG(17:1+18:3+16:0) | **2.58E-05** | 3.91 | 2.91E-01 | 0.60 | 5.20E-01 | 1.60 |
| **TG(52:2)** | TG(16:0+18:1+18:1) | **4.97E-04** | 1.61 | 7.92E-02 | 0.67 | 3.08E-01 | 1.63 |
| **TG(52:3)** | TG(16:0+18:1+18:2) | **1.54E-04** | 2.33 | 1.01E-01 | 0.72 | 4.34E-01 | 1.57 |
| **TG(52:4)** | TG(16:0+18:2+18:2) | **2.48E-05** | 4.34 | 1.48E-01 | 0.72 | 5.02E-01 | 1.74 |
| **TG(52:5)** | TG(16:0+18:2+18:3) | **1.13E-05** | 3.66 | 3.02E-01 | 0.62 | 4.51E-01 | 2.00 |
| **TG(53:2)** | TG(18:1+18:1+17:0) | **1.41E-04** | 1.64 | 2.03E-01 | 0.66 | 4.91E-01 | 1.38 |
| **TG(53:3)** | TG(18:2+18:1+17:0) + TG(18:1+18:1+17:1) | **2.43E-05** | 2.49 | 2.60E-01 | 0.64 | 5.51E-01 | 1.43 |
| **TG(53:4)** | TG(18:2+17:1+18:1) | **7.51E-05** | 3.35 | 2.48E-01 | 0.58 | 5.39E-01 | 1.64 |
| **TG(54:1)** | TG(18:0+18:1+18:0) | 1.85E-01 | 0.76 | **1.35E-02** | 0.69 | 5.49E-01 | 2.05 |
| **TG(54:2)** | TG(18:0+18:1+18:1) | **2.57E-02** | 1.33 | **9.71E-03** | 0.60 | 5.46E-01 | 1.65 |
| **TG(54:3)** | TG(20:2+20:1+14:0) + TG(20:2+18:1+16:0) + TG(20:1+18:2+16:0) + TG(18:2+18:1+18:0) | **7.35E-04** | 2.17 | 9.97E-02 | 0.67 | 4.48E-01 | 1.61 |
| **TG(54:4)** | TG(18:2+18:1+18:1) + TG(18:2+18:2+18:0) | **3.04E-04** | 2.85 | 1.21E-01 | 0.74 | 5.03E-01 | 1.61 |
| **TG(54:5)** | TG(18:2+18:2+18:1) | **3.96E-05** | 4.53 | 1.34E-01 | 0.82 | 5.47E-01 | 1.64 |
| **TG(54:6)** | TG(18:2+18:3+18:1) | **5.98E-05** | 4.92 | 3.02E-01 | 0.81 | 5.10E-01 | 2.46 |
| **TG(54:6)** | TG(20:4+18:2+16:0) | **3.15E-02** | 1.80 | 2.73E-01 | 0.68 | 8.15E-01 | 1.15 |
| **TG(54:7)** | TG(18:2+18:3+18:2) | **4.09E-03** | 4.03 | 4.95E-01 | 1.31 | 4.66E-01 | 2.83 |
| **TAG(54:7)** | TG(20:5+18:2+16:0) | **1.01E-02** | 4.07 | 4.12E-01 | 1.40 | 3.85E-01 | 3.20 |
| **TG(56:2)** | TG(18:1+20:1+18:0) | 5.56E-01 | 0.94 | **1.83E-03** | 0.64 | 5.98E-01 | 1.54 |
| **TG(56:3)** | TG(18:1+20:1+18:1) | **5.97E-03** | 1.64 | **5.30E-06** | 0.59 | 5.94E-01 | 1.36 |
| **TG(56:5)** | TG(22:5+18:0+16:0) + TG(22:4+18:1+16:0) | **5.99E-03** | 1.59 | **3.39E-04** | 0.70 | 9.44E-01 | 1.04 |
| **TG(56:7)** | TG(22:5+18:2+16:0) + TG(20:4+18:2+18:1) | **6.01E-04** | 2.96 | 6.12E-01 | 0.85 | 5.67E-01 | 1.91 |
| **TG(56:7)** | TG(22:6+18:1+16:0) | **4.56E-04** | 3.96 | **3.47E-02** | 1.52 | 5.01E-01 | 2.41 |
| **TG(58:2)** | TG(18:1+22:0+18:1) | 9.67E-02 | 0.66 | **4.37E-02** | 0.54 | 5.66E-01 | 1.64 |
| **TG(58:3)** | TG(22:1+18:1+18:1) + TG(22:1+18:2+18:0) | 9.99E-02 | 0.78 | **1.79E-05** | 0.65 | 8.44E-01 | 1.12 |
| **TG(58:4)** | TG(22:0+18:2+18:2) | 1.64E-01 | 1.18 | **7.32E-03** | 0.65 | 9.29E-01 | 1.04 |
| **TG(58:6)** | TG(22:5+18:0+18:1) | **3.37E-02** | 1.30 | **8.07E-03** | 0.65 | 9.64E-01 | 0.97 |
| **TG(58:7)** | TG(22:5+18:2+18:0) + TG(22:4+18:2+18:1) + TG(22:5+18:1+18:1) | **5.19E-03** | 1.78 | 9.06E-01 | 0.98 | 7.32E-01 | 1.41 |
| **TG(58:8)** | TG(22:5+18:2+18:1) + TG(22:6+18:1+18:1) | **1.70E-04** | 3.21 | **6.52E-03** | 1.78 | 5.64E-01 | 1.99 |
| **TG(58:9)** | TG(20:4+20:4+18:1) + TG(22:6+18:1+18:2) | **4.18E-03** | 6.60 | **5.76E-03** | 1.92 | 4.65E-01 | 3.82 |
| **TG(58:10)** | TG(20:5+20:4+18:1) + TG(20:4+20:4+18:2) | **5.53E-03** | 4.86 | **6.18E-03** | 2.38 | 4.56E-01 | 4.09 |

| **PHOSPHATIDYLCHOLINE** | | | | | | | |
| --- | --- | --- | --- | --- | --- | --- | --- |
| ***Individual notation*** | ***Individual composition or probable ID*** | ***p-value*** | ***Fold change*** | ***p-value*** | ***Fold change*** | ***p-value*** | ***Fold change*** |
|  |  | ***OPN-KO vs WT Quiescent*** | | ***OPN-KO vs WT 24 h*** | | ***OPN-KO vs WT 48 h*** | |
| **PC(14:0/20:4)** |  | **3.77E-03** | 0.70 | **2.61E-02** | 0.81 | 2.18E-01 | 0.86 |
| **PC(15:0/20:4)** |  | **4.78E-02** | 0.78 | 5.06E-01 | 0.93 | 3.82E-01 | 0.75 |
| **PC(15:0/22:6)** |  | **1.72E-02** | 1.37 | 5.83E-01 | 1.12 | 4.16E-01 | 0.75 |
| **PC(16:0/16:0)** |  | **2.11E-02** | 0.78 | 7.29E-01 | 0.98 | 2.51E-01 | 0.92 |
| **PC(16:0/17:0)** |  | **2.40E-02** | 0.52 | 7.46E-01 | 0.90 | 7.19E-01 | 1.10 |
| **PC(16:0/18:0)** |  | **1.19E-02** | 0.66 | 2.25E-01 | 0.91 | **3.93E-02** | 0.84 |
| **PC(16:0/18:1)** |  | **5.50E-02** | 0.71 | 5.33E-01 | 0.95 | 2.39E-01 | 1.16 |
| **PC(16:0/20:4)** |  | 1.57E-01 | 0.89 | **4.08E-02** | 0.89 | 7.22E-02 | 0.93 |
| **PC(16:0/20:5)** |  | 3.66E-01 | 1.21 | **5.73E-03** | 2.52 | **1.00E-02** | 1.91 |
| **PC(17:0/20:4)** |  | **2.85E-02** | 0.67 | 8.81E-01 | 1.04 | 3.91E-01 | 0.77 |
| **PC(18:0/18:1)** |  | **1.21E-02** | 0.69 | 1.13E-01 | 0.77 | 3.78E-01 | 0.91 |
| **PC(18:0/18:2)** |  | **4.58E-02** | 0.86 | 3.23E-01 | 0.89 | 1.64E-01 | 0.91 |
| **PC(18:0/20:3)** |  | **5.15E-02** | 0.80 | 5.16E-01 | 0.90 | 6.13E-01 | 1.07 |
| **PC(18:0/20:4)** |  | 2.19E-01 | 0.86 | **3.06E-02** | 0.82 | **4.71E-02** | 0.79 |
| **PC(18:0/22:4)** |  | **1.88E-03** | 0.65 | **3.91E-04** | 0.60 | **1.30E-05** | 0.62 |
| **PC(20:0/18:2)** |  | **4.26E-03** | 0.75 | 4.04E-01 | 0.86 | **4.40E-02** | 0.83 |
| **PC(20:0/20:4)** |  | 2.59E-01 | 0.84 | 9.85E-02 | 0.76 | **6.66E-04** | 0.76 |
| **PC(31:0)** |  | **4.32E-02** | 0.80 | 4.46E-01 | 0.93 | 4.18E-01 | 0.88 |
| **PC(33:1)** | PC(15:0/18:1) + PC(16:0/17:1) | **4.93E-02** | 0.74 | 1.03E-01 | 0.88 | 7.58E-01 | 1.11 |
| **PC(38:5)** | PC(18:1/20:4) + PC(16:0/22:5) | 2.45E-01 | 0.87 | 5.59E-01 | 0.95 | **2.39E-04** | 0.84 |
| **PC(38:5)** | PC(18:0/20:5) + PC(16:0/22:5) | **4.81E-02** | 0.72 | 9.30E-01 | 1.01 | 9.32E-02 | 0.77 |
| **PC(40:5)** |  | **1.62E-03** | 0.61 | **1.22E-02** | 0.54 | **3.45E-04** | 0.53 |
| **PC(18:1e/20:4)** |  | **7.83E-03** | 0.70 | 5.15E-02 | 0.81 | 5.63E-02 | 0.83 |
| **PC(18:1e/22:4)** |  | **1.14E-02** | 0.63 | **5.32E-03** | 0.65 | **3.87E-02** | 0.51 |
| **PC(34:0e)** | PC(O-16:0/18:0) + PC(O-18:0/16:0) | **1.03E-03** | 0.61 | 1.05E-01 | 0.76 | **4.65E-02** | 0.78 |
| **PC(34:1e)** | PC(O-16:0/18:1) + PC(18:1e/16:0) | **3.56E-03** | 0.69 | 3.26E-01 | 0.86 | 1.01E-01 | 0.79 |
| **PC(O-16:0/16:0)** |  | **3.56E-02** | 0.78 | 8.10E-01 | 1.07 | 3.04E-01 | 0.88 |
| **PC(O-16:0/20:4)** |  | **1.69E-02** | 0.74 | 7.97E-02 | 0.83 | 1.27E-01 | 0.83 |
| **PC(O-16:0/22:4)** |  | **2.27E-02** | 0.68 | **1.41E-02** | 0.68 | 5.26E-02 | 0.61 |
| **PC(O-18:0/20:4)** |  | **1.00E-02** | 0.69 | **5.28E-03** | 0.61 | **1.41E-02** | 0.64 |
| **PC(O-20:0/20:4)** |  | 6.78E-02 | 0.73 | 1.05E-01 | 0.60 | **1.33E-04** | 0.50 |
| **PC(14:0/0:0)** |  | **1.33E-03** | 0.65 | 3.35E-01 | 0.90 | 6.95E-01 | 0.94 |
| **PC(18:0/0:0)** |  | **6.40E-04** | 0.82 | **3.75E-02** | 0.87 | **2.07E-02** | 0.90 |
| **PC(18:3/0:0)** |  | 7.30E-01 | 0.96 | 8.90E-01 | 0.97 | **3.06E-02** | 1.32 |
| **PC(18:3/0:0)** |  | **2.48E-02** | 0.76 | 7.26E-01 | 0.80 | 9.00E-01 | 1.02 |
| **PC(20:0/0:0)** |  | **3.39E-03** | 0.79 | **9.20E-03** | 0.77 | **1.81E-02** | 0.81 |
| **PC(20:1/0:0)** |  | **7.67E-03** | 0.70 | **7.08E-03** | 0.74 | 2.45E-01 | 0.88 |
| **PC(20:4/0:0)** |  | **7.63E-03** | 0.74 | 1.88E-01 | 0.85 | 6.29E-02 | 0.71 |
| **PC(20:5/0:0)** |  | 8.87E-01 | 0.97 | **5.68E-03** | 1.98 | 2.15E-01 | 1.40 |
| **PC(22:4/0:0)** |  | **2.16E-03** | 0.59 | **3.42E-02** | 0.68 | **2.10E-02** | 0.48 |
| **PC(22:5/0:0)** |  | 6.33E-01 | 1.07 | **3.50E-02** | 1.52 | 2.53E-01 | 0.75 |
| **PC(22:5/0:0)** |  | 7.28E-02 | 0.56 | 1.30E-01 | 0.47 | **7.57E-03** | 0.37 |
| **PC(0:0/14:0)** |  | **4.13E-03** | 0.68 | 3.61E-01 | 0.92 | 4.07E-01 | 0.85 |
| **PC(0:0/15:0)** |  | **4.16E-02** | 0.76 | 8.11E-01 | 1.03 | 2.63E-01 | 0.70 |
| **PC(0:0/16:0)** |  | **2.57E-02** | 0.80 | 5.60E-01 | 0.96 | 1.20E-01 | 0.80 |
| **PC(0:0/18:0)** |  | **7.76E-03** | 0.69 | 4.11E-01 | 0.87 | **1.60E-02** | 0.72 |
| **PC(0:0/18:1)** |  | **3.64E-02** | 0.81 | 1.57E-01 | 0.88 | 8.92E-01 | 0.98 |
| **PC(0:0/20:1)** |  | 1.47E-01 | 0.81 | 2.61E-01 | 0.87 | **4.93E-02** | 0.77 |
| **PC(0:0/20:2)** |  | **4.19E-02** | 0.78 | 5.94E-01 | 0.94 | 2.31E-01 | 0.87 |
| **PC(0:0/20:3)** |  | 6.55E-01 | 0.95 | 9.87E-01 | 1.00 | **1.94E-02** | 1.29 |
| **PC(0:0/20:4)** |  | **3.53E-02** | 0.86 | 5.65E-02 | 0.84 | 5.51E-01 | 0.96 |
| **PC(0:0/20:5)** |  | 6.58E-01 | 1.10 | **9.64E-03** | 2.06 | **1.03E-02** | 1.89 |
| **PC(0:0/22:4)** |  | 1.83E-01 | 0.77 | 3.89E-01 | 0.83 | **3.12E-02** | 0.66 |
| **PC(0:0/22:5)** |  | 3.22E-01 | 0.79 | **2.72E-02** | 0.63 | **1.12E-02** | 0.54 |
| **PC(18:1e/0:0)** |  | **3.46E-03** | 0.68 | **5.83E-03** | 0.80 | 1.25E-01 | 0.87 |
| **PC(18:1e/0:0)** |  | 1.01E-01 | 0.79 | 3.63E-01 | 1.07 | **1.43E-02** | 0.65 |
| **PC(O-16:0/0:0)** |  | **7.91E-03** | 0.80 | 6.85E-01 | 0.97 | **3.81E-02** | 0.87 |
| **PC(O-18:0/0:0)** |  | **3.50E-02** | 0.83 | **6.99E-03** | 0.78 | **2.32E-03** | 0.73 |
| **PC(O-20:0/0:0)** |  | 6.82E-01 | 0.94 | 2.87E-01 | 0.84 | **3.12E-02** | 0.71 |

Lipidomic analysis of livers from *OPN-KO* mice was performed and compared with that of their corresponding WT animals. “Individual notation” refers to the confirmed identification of the metabolites. Overlapping of two o more metabolites or non-confirmed identification is indicated in “Individual composition (or probable ID)”. Fold-changes and unpaired Student’s t test *p*-values (or Welch´s t test where unequal variances were found) were calculated for each comparison considering 5 animals per group. Significant differences are in bold letters.

**Supplemental Figure 1**

**Suplementary Figure 1. Atorvastatin treatment induces the same ROS production in OPN-KO and WT mice during liver regeneration.** Wild type (WT) and osteopontin knockout (OPN-KO) treated with atorvastatin (100 mg/kg) mice were subjected to partial hepatectomy (PH) and livers slices from quiescent, 24 h and 48 h post-hepatectomy were collected. (A) Representative liver sections stained with DHE (left) and quantification (right). (B) Liver levels of specific oxidated fatty acids were measured: oxo-octadecadenoic acids (X-Oxo ODE), Hydroxy-octadecadenoic acids (X-H ODE), Hydroxy-eicosatetraenoic acids (X-H ETE).

**Supplemental Figure 2**

**Suplementary Figure 2. Atorvastatin treatment maintained unaltered Ki67 in OPN deficient mice.** Osteopontin knockout mice (OPN-KO) treated with atorvastatin (100 mg/kg) or vehicle were subjected to partial hepatectomy (PH), and livers from 48 h post-PH were collected and Ki67 immunostaining was performed.

**Supplemental Figure 3**

**Suplementary Figure 3. Atorvastatin treatment does not affect regeneration in WT mice 24 and 48 h post-hepatectomy.** Wild type (WT) treated with atorvastatin (100 mg/kg) mice were subjected to partial hepatectomy (PH) and livers sections from 24 h and 48 h post-hepatectomy were collected. (A) The percentage of liver weight to body weight was calculated. (B) Representative liver sections stained with Ki67 (right) and quantification (left).

**Complete Western Bloting Images**

From Figure 1:

From Figure 4:


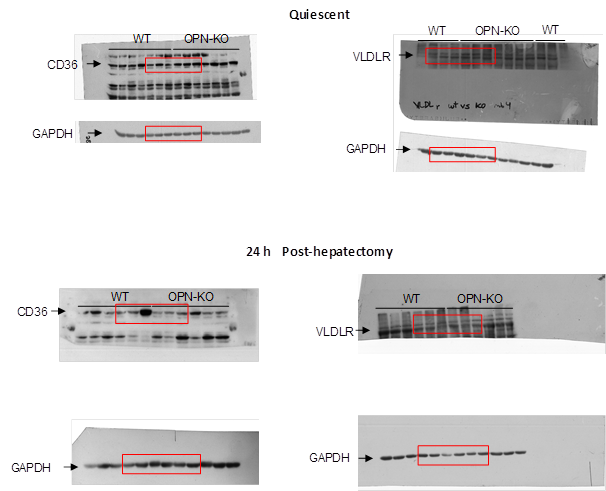

Supplement: Supplementary file 1 — Supplementary Information [file 41598_2018_32919_MOESM1_ESM.doc]
